# Supplementary material for: Identification of photoperiod-induced specific miRNAs in the adrenal glands of Sunite sheep (Ovis aries)
Source: Front Vet Sci. 2022 Jul 22;9:888207. doi: 10.3389/fvets.2022.888207 (PMC9354845; doi:10.3389/fvets.2022.888207)
Supplement: Supplementary file 1 [file Data_Sheet_1.ZIP › Supplementary file/miRDeep2 file.zip!/miRDeep2 file/Novel_Structure/LP42A1/Novel_271.pdf]

Provisional ID	:	NC_019466.2_17332
Score total	:	0
Score for star read(s)	:	-1.3
Score for read counts	:	-1.8
Score for mfe	:	1.5
Score for randfold	:	1.6
Score for cons. seed	:	
Total read count	:	8
Mature read count	:	7
Loop read count	:	0
Star read count	:	1

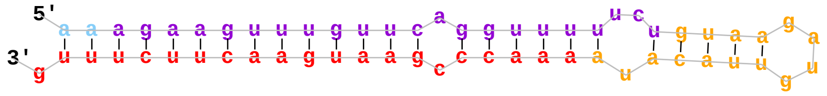

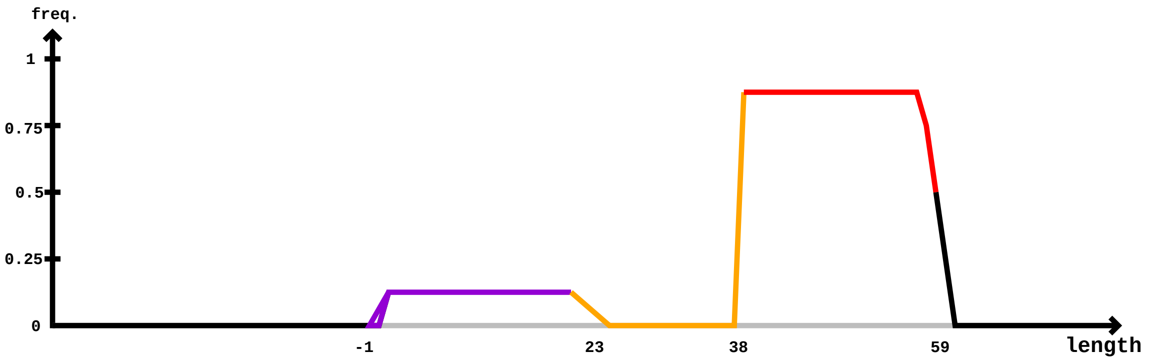


```
                                    Star                                    Mature
5'- guguuuaaugaaagaauauauugggguggcaaaagaaguuuguucagguuuuucuguaagauguuacauaaaacccgaaugaacuucuuugcucaaccuaauagaacuu  -3'  obs
    guguuuaaugaaagaauauauugggguggcaaaagaaguuuguucagguuuuucuguaagauguuacauaaaacccgaaugaacuucuuugcucaaccuaauagaacuu       exp
    ...........(((....(((((((.(((((((.(((((((((((.((((((..(((((....))))).)))))).))))))))))))))))).)).)))))))...)))  reads  mm  sample
    .................................aAaaguuuguucagguuuuucu......................................................  1      1   seq
    ......................................................................aaacccgaaugaacuucuu....................  1      0   seq
    ......................................................................aaacccgaaugaacuuUuuu...................  2      1   seq
    ......................................................................aaacccgaaugaacuuUuuug..................  4      1   seq
```
